# Therapeutic Effect of Shikimic Acid on Heat Stress-Induced Myocardial Damage: Assessment via Network Pharmacology, Molecular Docking, Molecular Dynamics Simulation, and In Vitro Experiments

**DOI:** 10.3390/ph17111485

**Published:** 2024-11-05

**Authors:** Yan Gu, Jingyi Zhang, Haohong Zheng, Yuyang Qin, Min Zheng, Yanchun Hu, Jialiang Xin

**Affiliations:** 1 Key Laboratory of Animal Disease and Human Health of Sichuan Province, College of Veterinary Medicine, Sichuan Agricultural University, Chengdu 611130, China; 18583246371@163.com (Y.G.); threemoonlight@163.com (J.Z.); haohongzheng916@gmail.com (H.Z.); yuyscu@163.com (Y.Q.); 2 Guangxi Center for Animal Disease Control and Prevention, Nanning 530001, China; zhgmn26@163.com

**Keywords:** network pharmacology, heat stress, myocardial damage, shikimic acid, molecular docking, molecular dynamics simulation

## Abstract

**Abstract:** Background: Rising global temperatures have been linked to an increased incidence of heat stress (HS)-induced myocardial damage. Methods: This study aimed to investigate the therapeutic potential of shikimic acid (SA) on HS-induced myocardial damage using network pharmacology, molecular docking, molecular dynamics (MD) simulations, and in vitro experiments. Results: Network pharmacology analysis indicated that SA significantly attenuates the inflammatory response to HS by modulating 60 targets, including TNF, IL-6, and STAT3, which are enriched in the PI3K/AKT signaling pathway. Molecular docking and MD simulation analyses demonstrated that SA forms stable complexes with TNF (−6.642 kcal/mol) and IL-6 (−7.261 kcal/mol), with no significant conformational changes over a 100 ns simulation period. In vitro experiments demonstrated that SA, within the concentration range of 250 μM to 31.25 μM, significantly promoted the proliferation of normal HL-1 cells by an average of 31.0%. Moreover, it enhanced the survival rate of HL-1 cells exposed to 43 °C for 3 h by approximately 59.9% and downregulated the expression of Hsp90 and Hsp70. Additionally, this concentration range of SA reduced the expression of TNF-α, IL-6, TLR2, and COL1A1. Conclusions: These findings offer evidence for the therapeutic potential of SA in HS-induced myocardial damage.

## 1. Introduction

Climate change presents a profound challenge to humanity in the 21st century, as evidenced by rising global surface temperatures and the increasing frequency of extreme heat events [1]. High temperatures can induce heat stress (HS), which poses a significant threat, particularly to vulnerable populations, and heightens the risk of cardiovascular mortality among individuals aged 65 and older [2,3]. Elevated temperatures lead to an increased myocardial oxygen demand while simultaneously impairing blood flow and oxygen delivery to the heart. This imbalance results in elevated production of reactive oxygen species (ROS) [4], which induces oxidative stress. Studies have demonstrated that oxidative stress activates various intracellular signaling pathways, including the p38 mitogen-activated protein kinase (MAPK) pathway [5], nuclear factor kappa B (NF-κB) signaling [6], and caspase cascades [7]. Currently, symptomatic management is the primary strategy for addressing HS-induced myocardial damage [8]. However, this approach is increasingly insufficient due to global heatwaves intensifying. Thus, more strategies are urgently needed to better mitigate the adverse effects of heat stress.

Shikimic acid (SA) is a natural organic compound derived from the seeds of the Chinese star anise plant (*Illicium verum*) [9,10]. Research has shown that SA significantly reduces oxidative stress markers in rat models of lactate diet-induced diarrhea, including superoxide dismutase (SOD), glutathione peroxidase (GSH-Px), catalase (CAT), malondialdehyde (MDA), NADPH oxidase activity (NOX), conjugated dienes (CD), and the oxidative stress index (OS) [11]. Recent studies indicate that SA modulates the p38 MAPK [12,13] and NF-κB [14,15] signaling pathways, reducing the expression of pro-inflammatory cytokines such as IL-1β, TNF-α, and IL-6, and exhibiting both anti-inflammatory and anti-apoptotic effects. Additionally, SA inhibits P-selectin expression on activated platelets, providing an anti-cardiovascular embolic effect [16]. While the specific efficacy of SA in treating HS has not been conclusively demonstrated, its pharmacological properties suggest potential effects in alleviating HS-induced myocardial damage.

**Figure 1:**
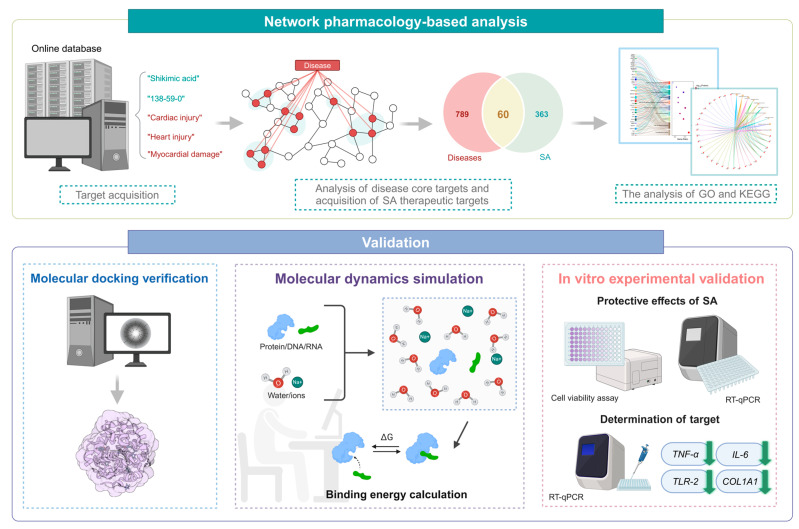
Flowchart of the study design.

Network pharmacology, molecular docking, and molecular dynamics simulation are essential methodologies for elucidating drug action mechanisms. Network pharmacology integrates drug-target-disease relationships to comprehensively understand therapeutic effects, predicting drug mechanisms and pathways from extensive datasets [17]. Molecular docking, a widely utilized computational technique, predicts the binding affinity and interaction modes between small molecules and target proteins, aiding in identifying potential therapeutic agents [18]. Molecular dynamics (MD) simulation further assesses the stability and dynamics of drug-target interactions under physiological conditions, enhancing our understanding of protein-ligand interactions and their functional impacts [19,20]. This study aims to use network pharmacology to identify potential molecular targets and signaling pathways related to the therapeutic effects of SA on HS-induced myocardial damage. Molecular docking, MD simulation, and in vitro experiments will be employed to validate these findings. Figure 1 illustrates the study’s methodology.

## 2. Results

### 2.1. Network Pharmacological Analysis

#### 2.1.1. Targets Acquisition of Disease

**Figure 2:**
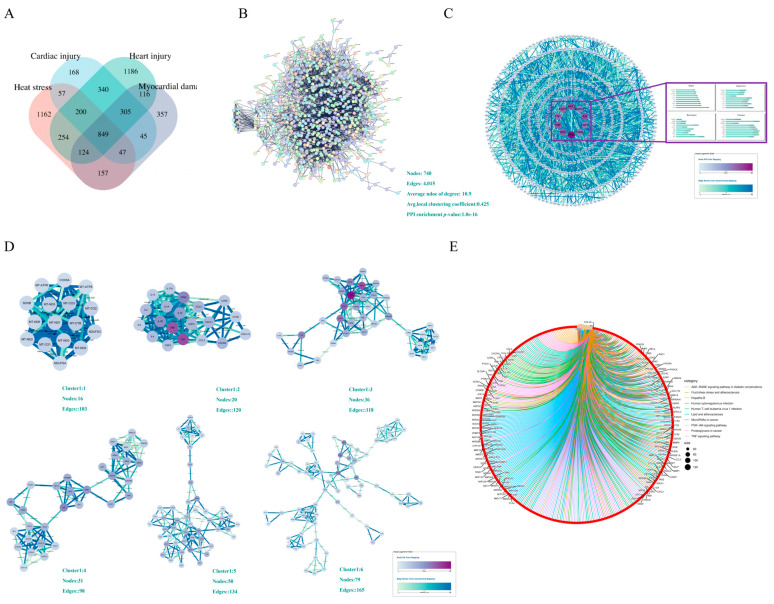
Analysis of HS-induced Myocardial damage. (**A**) The Venn analysis of common targets between “heat stress” and “myocardial damage”, as well as between “heart injury” and “cardiac injury”. (**B**) PPI network of the disease-related targets. (**C**) Analysis of core targets of disease by analysis of 4 algorithms using Cytoscape 3.9.1 software. (**D**) The functional clusters in disease-related targets by MCODE analysis using Cytoscape. (**E**) The KEGG analysis of disease-related targets.

The number of targets retrieved based on keywords in each database is presented in Table 1. For datasets exceeding 2000 targets, only the top 2000 are included in the analysis, while all targets from datasets with fewer than 2000 are considered. Following these criteria, 2011 targets were identified using “cardiac injury” as the keyword, while 3373 targets were collected with “heart injury”. Additionally, 2000 targets were retrieved using “myocardial damage”, and 2850 targets were obtained with “heat stress”. Venn diagram analysis revealed 849 common targets between “heat stress” and “myocardial damage”, as well as between “heart injury” and “cardiac injury” (Figure 2A).

#### 2.1.2. PPI Network Construction

Disease-related targets were input into STRING version 12.0, focusing exclusively on Homo sapiens with an interaction score greater than 0.9. This generated a protein–protein interaction (PPI) network consisting of 4015 edges and 740 nodes (Figure 2B). The disease-related target PPI network was then imported into Cytoscape 3.9.1, and four algorithms (Betweenness, Closeness, Degree, Eigenvector) were utilized via the CytoNCA plugin in Cytoscape 3.9.1 to identify core targets. The analysis revealed that the top ten core targets were TP53, SRC, TNF, FN1, STAT3, AKT1, MAPK1, IL-6, EGFR, and CTNNB1 (Figure 2C). Additionally, six clusters were identified with scores exceeding 4 (Figure 2D). The Kyoto Encyclopedia of Genes and Genomes (KEGG) analysis indicated that the primary pathogenic pathways associated with HS-induced myocardial damage included the AGE-RAGE signaling pathway in diabetic complications, lipid metabolism, and atherosclerosis, and the PI3K/AKT signaling pathway (Figure 2E).

#### 2.1.3. The Common Target Analysis

A total of 285 potential targets were identified from the PharmMapper Server. The CTD added 9 additional targets, while STRING provided 33. The TCMSP contributed 5 targets, and the Swiss Target Prediction yielded 100. No targets were identified from the Binding Database or the TTD. After consolidating the data and removing duplicates, we obtained 423 targets for SA.

**Figure 3:**
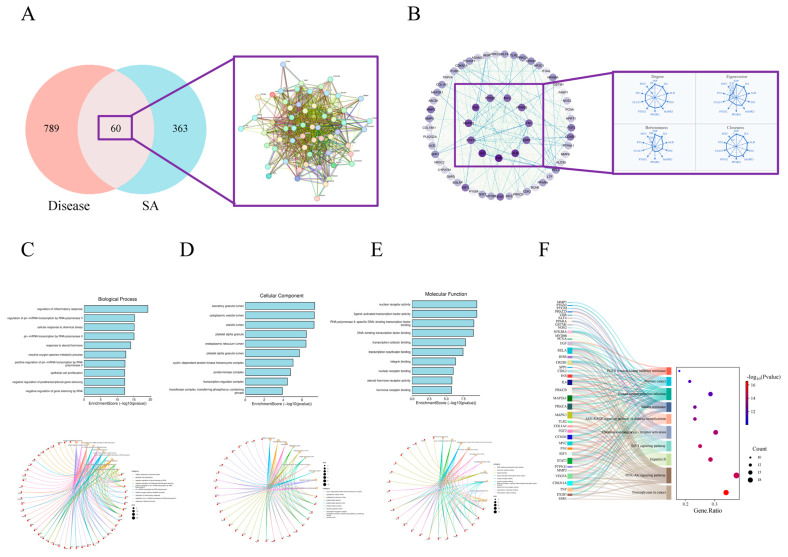
Analysis of common targets between SA and disease. (**A**) the Venn analysis of disease-related targets and SA targets. (**B**) Analysis of core targets of common targets between SA and disease by analysis of 4 algorithms using Cytoscape 3.9.1 software. (**C**) Enrichment analysis of biological process of 60 common targets. (**D**) Enrichment analysis of cellular component of 60 common targets. (**E**) Enrichment analysis of molecular function of 60 common targets. (**F**) KEGG pathway analysis of 60 common targets.

The Venn analysis identified 60 overlapping targets between SA and disease-related targets (Figure 3A). These overlapping targets were determined using four different algorithms, consistently indicating that TNF, IL-6, and STAT3 are core targets through which SA impacts disease (Figure 3B). To further predict the specific effects of SA on diseases and the pathways involved, as well as diseases and the associated pathways, we utilized the bioinformatics tool DAVID for Gene Ontology (GO) and KEGG. In the GO analysis, we identified 76 biological processes (BP), 25 cellular components (CC), and 33 molecular function (MF) items from a dataset of 849 disease-related targets. Significant biological terms included the regulation of primiRNA transcription by RNA polymerase II, modulation of the inflammatory response, cellular stress response, and steroid hormone response (Figure 3C). Key categories identified in the cellular component analysis were the endoplasmic reticulum lumen, platelet alpha granule, vesicular lumen, cytoplasmic vesicular lumen, and secretory granule lumen (Figure 3D). Regarding molecular function, critical terms included DNA-binding transcription factor activity specific to RNA polymerase II, nuclear receptor activity, ligand-activated transcription factor activity, and general DNA-binding transcription factor binding (Figure 3E). Details are presented in Supplementary Table S1.

Additionally, KEGG enrichment analysis revealed 230 disease-related targets, indicating that SA can influence disease mechanisms by regulating the AGE-RAGE signaling pathway in diabetic complications and the PI3K-Akt signaling pathway (Figure 3F). Details are presented in Supplementary Table S1.

### 2.2. Molecular Docking

**Figure 4:**
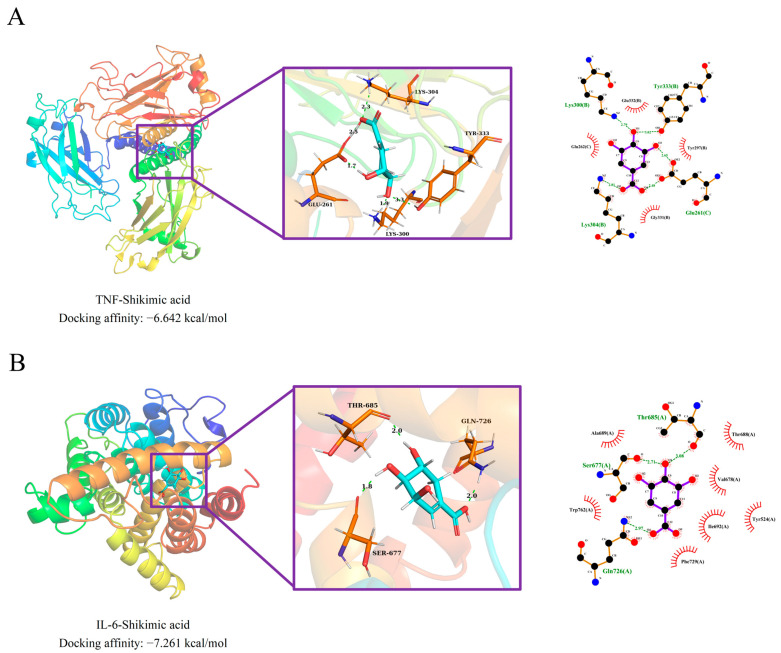
Molecular docking results. (**A**) Visualization of TNF docking with shikimic acid. (**B**) Visualization of IL-6 docking with shikimic acid.

To confirm whether SA can interfere with the pathogenic targets of HS-induced myocardial damage, we selected IL-6 and TNF for molecular docking with SA, as these are key targets of SA in disease modulation. Molecular docking results indicated that SA (PubChem ID: 8742) stably binds to TNF (PDB ID: 5E1T) and IL-6 (PDB ID: 5SFK) with docking affinities of −6.642 kcal/mol and −7.261 kcal/mol, respectively (Figure 4A,B). Further analysis revealed that SA forms hydrogen bonds with specific amino acid residues in TNF, including LYS304, GLU261, LYS300, and TYR333, while also engaging in hydrophobic interactions with surrounding residues (Figure 4A). Similarly, SA establishes hydrogen bonds with THR685, GLN726, and SER677 in IL-6 alongside hydrophobic interactions with neighboring residues (Figure 4B). These findings suggest that SA may interfere with the IL-6 and TNF, potentially mitigating HS-induced myocardial damage.

### 2.3. MD Simulation

#### 2.3.1. Stability of TNF-Shikimic Acid and IL-6-Shikimic Acid Complexes

**Figure 5:**
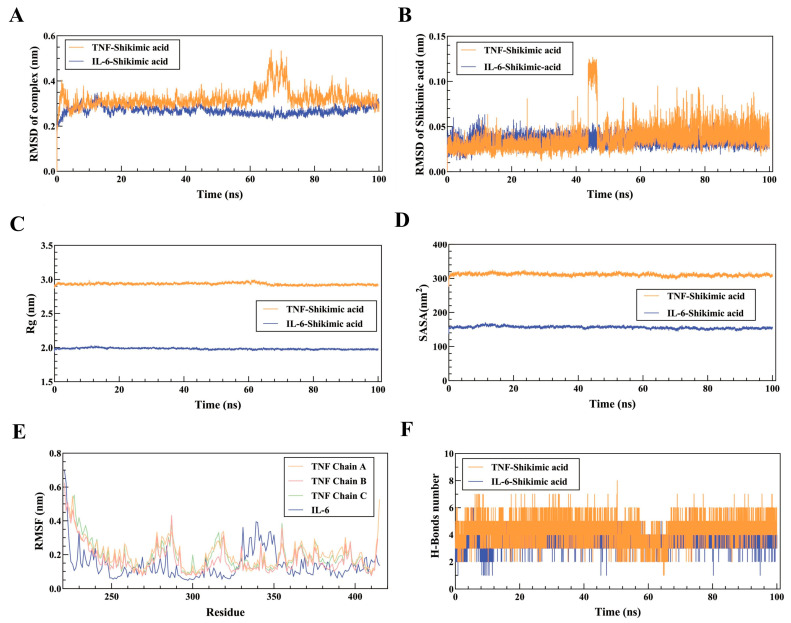
Analysis of 100 ns molecular dynamics simulations of TNF-SA and IL-6-SA complexes. (**A**) RMSD curves of TNF-SA and IL-6-SA complexes, (**B**) RMSD curves of SA in the TNF-SA and IL-6-SA complex system, (**C**) TNF-SA and IL-6-SA complex Rg curves, (**D**) SASA curves of TNF-SA and IL-6-SA complexes, (**E**) RMSF curves of TNF-SA and IL-6-SA complexes, (**F**) RMSF curves of TNF-SA and IL-6-SA complexes hydrogen bonding change curves.

To further assess the impact of SA on IL-6 and TNF proteins, MD simulation analysis was utilized to ascertain the stability of complexes in 100 ns. The root-mean-square deviation (RMSD) curve for the TNF-SA complex showed significant fluctuations between 60 and 70 ns but remained stable otherwise, with an average RMSD of 0.3 nm (Figure 5A). The IL-6-SA complex reached equilibrium after 20 ns, with an average RMSD of 0.26 nm (Figure 5A). The stable RMSD curve of SA in TNF throughout the simulation indicates a stable binding site. Meanwhile, the RMSD curve for SA in IL-6 showed minor fluctuations between 45 and 50 ns before stabilizing (Figure 5B). The radius of gyration (Rg) curves for both TNF- and IL-6-SA complexes remained stable, with average Rg values of 2.9 nm and 2.0 nm, respectively (Figure 5C). Moreover, the solvent-accessible surface area (SASA) curves of TNF-SA and IL-6-SA complexes also remained stable, with average SASA values of 310 nm^2^ and 160 nm^2^, respectively (Figure 5D). The Root Mean Square Fluctuation (RMSF) analysis indicated that there was no significant change in amino acid flexibility in the TNF-SA complex system. In contrast, amino acids in the IL-6-SA complex exhibited increased flexibility in the amino acid region from 330 to 350 (Figure 5E). The analysis of H-bond formation revealed the TNF-SA complex formed between 1 and 8 H-bonds and the IL-6-SA complex formed between 1 and 6 H-bonds (Figure 5F).

#### 2.3.2. Gibbs Free Energy Analysis and Free Energy (MM/PBSA) Analysis

**Figure 6:**
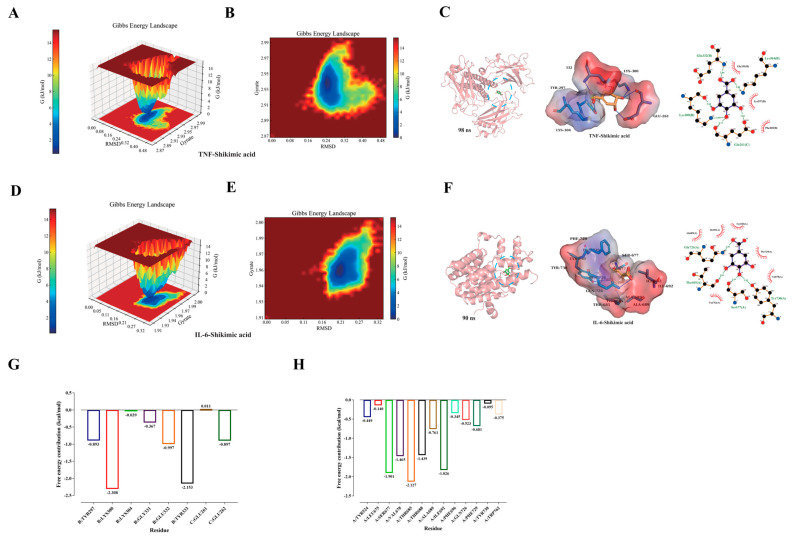
Gibbs free energy analysis and free energy (MM/PBSA) analysis. (**A**) Gibbs free energy 3D diagram of TNF-SA complex. (**B**) Gibbs free energy 2D diagram of TNF-SA complex. (**C**) Conformational analysis of TNF-SA complex at the lowest Gibbs energy moment. (**D**) Gibbs free energy 3D diagram of IL-6-SA complexes. (**E**) Gibbs free energy 2D diagram of IL-6-SA complexes. (**F**) Conformational analysis of IL-6-SA complex at the lowest Gibbs energy moment. (**G**) Amino acid decomposition of TNF-SA complex. (**H**) Amino acid decomposition of IL-6-SA complex.

The built-in scripts “g_sham” and “xpm2txt.py” from Gromacs 2022.03 were used to calculate the Gibbs free energy about the RMSD and Rg values of the IL-6-SA and TNF-SA complexes. The Gibbs free energy diagram was employed to elucidate the stability of receptor-ligand interactions. The stable conformation of the complex is most evident in the lowest free energy regions, indicated by blue and purple areas, which represent lower energies. A free energy landscape with multiple minimum energy clusters and rough surfaces suggests weak or unstable interactions between protein and ligand. In contrast, strong and stable interactions result in nearly singular, smooth energy clusters. The two-dimensional (Figure 6A) and three-dimensional (Figure 6B) representations of the TNF-SA complex revealed a single, distinct minimum energy region, as per the Gibbs free energy calculations. Figure 6C illustrates the lowest Gibbs energy region of the TNF-SA complex. Similarly, the IL-6-SA complex showed a single, distinct minimum energy region in both 3D (Figure 6D) and 2D (Figure 6E) morphologies. The visualization of the lowest Gibbs energy region for the IL-6-SA complex is depicted in Figure 6F. Moreover, the free energy stability between complexes was quantitatively assessed using MM/PBSA analysis. The TNF-SA complex exhibited a total binding free energy of −21.79 ± 1.08 kJ/mol, whereas the IL-6-SA complex had a total binding free energy of −18.67 ± 2.88 kJ/mol. Detailed data can be found in Table 2. This study also conducted a decomposition analysis of the amino acids in the binding pockets of the TNF-SA and IL-6-SA complexes to identify critical residues contributing to the binding free energy. A total of 8 and 13 residues were significant for the TNF- and IL-6-SA complexes, respectively (Figure 6G,H). For the TNF-SA complex, LYS300 (B chain) and TYR333 (B chain) were the most influential. In the case of the IL-6-Shikimic acid complex, SER677 (A chain), VAL678 (A chain), THR685 (A chain), THR688 (A chain), and ILE692 (A chain) were the most significant contributors.

### 2.4. In Vitro Experimental Validation

#### 2.4.1. SA Protects HL-1 Cells from HS

**Figure 7:**
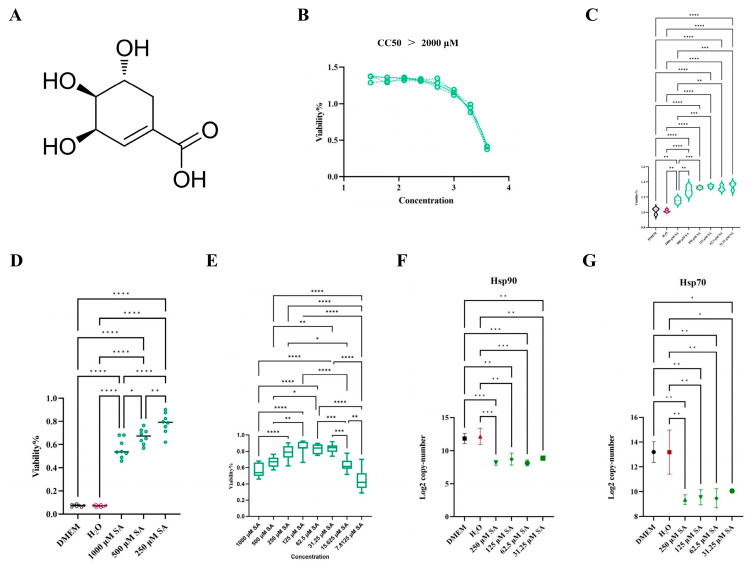
Validation by In vitro experiments. (**A**) The chemical formula of SA. (**B**) CC50 of SA in HL-1 cells. (**C**) The effect of SA on HL-1 cell proliferation was determined by CCK-8. (**D**) Protective effect of SA on HL-1 cells treated at 43 °C for 3 h. (**E**) Comparison of effects of different concentrations of SA on heat stress damage. (**F**) The expression of Hsp90. (**G**) The expression of Hsp70. All values represent the mean ± SD. **** *p* < 0.0001, *** *p* < 0.001,** *p* < 0.01, * *p* < 0.05.

The chemical structure of SA is shown in Figure 7A. To determine the non-cytotoxic concentration of SA in HL-1 cells, the cells cultured in 96 plates were exposed to 4000 μM, 2000 μM, 1000 μM, 500 μM, 125 μM, 62.5 μM, and 31.25 μM of SA, respectively. CCK-8 was utilized to measure the viability of HL-1 cells exposure to six different SA concentrations for 24 h. The results revealed that 1000 μM is the maximum safe concentration of SA in HL-1 cells, with the CC50 value exceeding 2000 μM (Figure 7B). Moreover, the CCK-8 assay results indicated that concentrations ranging from 250 μM to 31.25 μM of SA significantly stimulated the proliferation of the normal HL-1 cells by an average of 31.0% (Figure 7C).

To assess the protective effect of SA on HL-1 cells subjected to HS, the CCK-8 was employed to measure the viability of HL-1 cells treated at 43 °C for 3 h. Additionally, the expression levels of Hsp90 and Hsp70 were determined by RT-qPCR. The findings indicated that the protective effect of SA on heat-stressed HL-1 cells showed a positive correlation with concentration (Figure 7D). Within the safe concentration range, 250 μM to 31.25 μM of SA enhanced the survival rate of HL-1 cells exposed to 43 °C for 3 h by approximately 59.9% (Figure 7E). The expression of Hsp90 and Hsp70 in heat-stressed HL-1 cells treated with SA was markedly decreased (Figure 7F,G).

#### 2.4.2. The Target Was Determined by RT-qPCR

**Figure 8:**
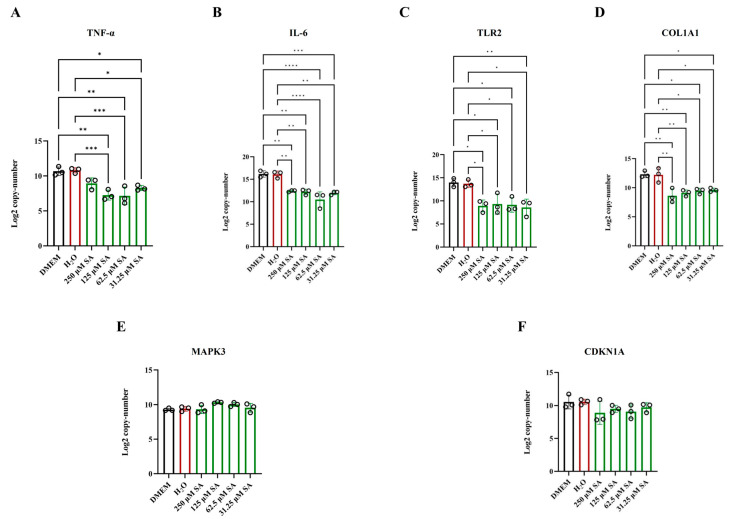
Target determination by RT-qPCR. (**A**) The expression of TNF-α. (**B**) The expression of IL-6. (**C**) The expression of TLR2. (**D**) The expression of COL1A1. (**E**) The expression of Mapk3. (**F**) The expression of CDKN1A. **** *p* < 0.0001, *** *p* < 0.001,** *p* < 0.01, * *p* < 0.05.

To substantiate the network pharmacological analysis findings, we examined the expression levels of IL-6 and TNF-α using RT-qPCR. Furthermore, to explore the role of SA in mitigating HS-induced myocardial damage via the PI3K/AKT signal pathway, we focused on targets known to be enriched in PI3K/AKT signaling pathway: COL1a1 (Figure 8A), IL-6 (Figure 8B), TLR2 (Figure 8C), COL1A1 (Figure 8D), MAPK3 (Figure 8E), and CDKNlA (Figure 8F). The gene expression analysis results indicated that SA significantly downregulates the expression of TNF-α, IL-6, TLR2, and COL1A1 in HL-1 cells subjected to HS for 3 h.

## 3. Discussion

The foundational concept of systems biology is the acknowledgment that the pathogenesis of complex diseases is often due to dysregulation within biological networks rather than defects in individual genes [21]. Network pharmacology, an interdisciplinary approach at the intersection of systems biology and pharmacology, enables researchers to more effectively identify and modulate the complex gene target networks in the body that are implicated in disease pathology. This strategy can potentially enhance the screening process for therapeutic interventions that can restore normal network function.

In this study, network pharmacology was employed to obtain the targets of HS-induced myocardial damage, and the protein–protein interaction (PPI) model was constructed. Based on the topological analysis of the PPI model based on 4 algorithms, TNF, IL-6, and IL-1β were identified as central targets in the pathogenesis of HS-induced myocardial damage. This suggests that HS triggers a robust inflammatory response in the myocardium. Subsequently, we performed MCODE analysis on the PPI network and discovered that these central targets are predominantly localized in the same subcluster. Modulation of these targets may effectively ameliorate the myocardial damage induced by HS. The results of KEGG enrichment analysis showed that the PI3K/AKT signaling pathway is the main pathway of HS-induced myocardial damage. These results are consistent with previous studies. It has been demonstrated that HS can produce a substantial amount of reactive oxygen species (ROS) within cells, resulting in the denaturation of mitochondrial proteins [22]. Such damage activates multiple pathways to produce inflammatory cytokines, such as the NLRP3/IL-1β/IL-6 pathway [23]. Although inflammatory factors represented by TNF-α, IL-1β, and IL-6 activate the PI3K/AKT signaling pathway to resist inflammation and promote cell survival and proliferation [24,25,26,27], the persistent elevation of these inflammatory cytokines, particularly TNF-α, IL-1β, and IL-6, can also induce sustained cell apoptosis and exacerbate tissue injury [28,29,30]. Thus, in treating myocardial damage caused by HS, it is essential to inhibit the expression of inflammatory factors.

This study identified 60 targets between HS-induced myocardial damage and SA through Venn analysis. These targets include many core targets of HS-induced myocardial damage, such as TNF, IL-6, STAT3, FN1, and IL-1β. GO enrichment analysis showed that SA can mainly correct the abnormal nuclear receptor activity in cardiomyocytes caused by HS and help cells restore regulation of the inflammatory response. Finally, the lumen of secretory granules and other cell components returned to normal. KEGG enrichment analysis showed that the PI3K/AKT signal pathway is the highest enrichment score for SA treatment of disease, which means SA may have the function of regulating this pathway. Molecular docking and molecular dynamics simulation were employed to assess the binding affinity of SA to bind to TNF and IL6, respectively. These techniques were utilized to evaluate the potential of SA to modulate these factors, thereby disrupting inflammatory pathways. The docking study revealed that SA exhibits stable binding to TNF and IL-6 with affinities of −6.642 kcal/mol and −7.261 kcal/mol, respectively. These values indicate a robust interaction between SA and both targets. Furthermore, MD simulations substantiated the stability of the TNF-SA and IL-6-SA complexes, demonstrating minimal conformational changes over a 100 ns timeframe. This suggests that once bound, the complexes maintain their integrity, reinforcing that SA can effectively engage with TNF and IL-6. In addition to molecular docking and molecular simulation techniques, we established in vitro HS damage models based on HL-1 cells and evaluated the protective effects of SA. The safety results showed that, although the maximum non-toxic dose of SA in HL-1 cells was 1000 μM, the treatment of HL-1 cells treated with HS was unsatisfactory. Administration of SA within the concentration range of 250 μM to 31.25 μM exerted the optimal protective effect on HL-1 cells exposed to 43 °C for 3 h, elevating cell survival by an average of approximately 59.9%. Pathological markers of HS, such as Hsp70 [31], Hsp90 [32], TNF-α [33], and IL-6 [34], were significantly reduced in HL-1 cells treated with SA in this range of concentrations. These pieces of evidence further confirm the role of SA in protecting against HS-induced myocardial damage.

Based on the KEGG analysis from network pharmacology in this and previous studies, IL-6, TLR2, COL1A1, MAPK3, and CDKN1A have been identified as key activators of the PI3K/AKT signaling pathway [35,36,37]. Our RT-qPCR results indicate that SA can significantly suppress the expression of Tlr2 and Col1ain HL-1 cells exposed to 43 °C for 3 h, except IL-6. This suggests that SA can inhibit the activation of the PI3K/AKT signaling pathway by inflammatory factors triggered by HS. Interestingly, while SA suppresses inflammatory factors that are typically associated with PI3K/AKT signaling pathway activation, it appears that under HS, this pathway may not be suppressed but could instead be activated to help cells combat inflammation. A previous study has shown that SA can activate the PI3K/AKT signaling pathway, thereby reducing cell apoptosis [38]. Consistent with this, our study found that SA concentrations ranging from 250 μM to 31.25 μM can effectively enhance cell viability by an average of 31.0% in healthy cells. The concentrations of SA that promote cell proliferation align with those that exhibit protective effects against HS-induced cell damage. Thus, we can reasonably hypothesize that SA can inhibit the rise in inflammatory factors caused by HS. Although SA reduces the levels of inflammatory factors and their activation of the PI3K/AKT signaling pathway, SA can activate the PI3K/AKT signaling pathway as a signaling molecule to assist cells in resisting inflammation and promoting cell proliferation.

## 4. Materials and Methods

### 4.1. Cell Lines and Compound

HL-1 cardiomyocytes procured from the China Center for Type Culture Collection (CCTCC) were cultured in Dulbecco’s modified Eagle’s medium (DMEM) (Gibico; 11965118; San Francisco, CA, USA) supplemented with 10% heat-inactivated fetal bovine serum (FBS) (Gibico; 30067334; San Francisco, CA, USA), 5000 U/mL of penicillin-streptomycin (Gibico; 15070063; San Francisco, CA, USA) at 37 °C in a 5% CO_2_ environment.

Shikimic acid (SA) with a purity ≥99.15% was purchased from MedChemExpress (CAS No.: 138-59-0) and diluted in H_2_O to achieve a final concentration of 100 mM.

### 4.2. Network Pharmacology Analysis

#### 4.2.1. Prediction of the SA Pharmacological Target

Targets associated with SA were identified by searching a variety of databases by using the keywords “Shikimic acid” and “138-59-0”, with a focus on Homo sapiens: TCMSPS (https://old.tcmsp-e.com/tcmsp.php, accessed on 11 April 2024), PharmMapper Server (http://www.lilab-ecust.cn/pharmmapper/, accessed on 11 April 2024), Swiss Target Prediction (http://www.swisstargetprediction.ch/, accessed on 11 April 2024) Binding Database (http://www.bindingdb.org/bind/index.jsp, accessed on 12 April 2024), STRING database (http://cn.string-db.org, accessed on 13 April 2024), CTD (https://ctdbase.org/, accessed on 13 April 2024), and TTD (http://db.idrblab.net/ttd/, accessed on 13 April 2024). Duplicate entries were eliminated, and the targets were combined once they were located using these sources.

#### 4.2.2. Collection of HS-Induced Myocardial Damage Related Targets

We collected HS targets from four databases: CTD (http://ctdbase.org, accessed on 14 April 2024), Disgenet (https://www.disgenet.org/, accessed on 14 April 2024), OMIM (https://omim.org/, accessed on 14 April 2024), and Genecards (https://www.genecards.org/, accessed on 14 April 2024), limited to Homo sapiens organisms, to identify the targets of HS-induced myocardial damage [39]. Then, we found targets related to myocardial damage in multiple databases by using the keywords “Myocardial damage”, “Heart injury”, and “Cardiac injury”. Based on the priority relevance ranking of the raw data, the top 2000 targets were selected, and duplicates were removed by keyword mergers. Finally, the HS-induced myocardial damage targets were obtained using JVenn (https://jvenn.toulouse.inrae.fr/app/example.html, accessed on 15 April 2024) to extract the common targets of HS-related targets and myocardial damage-related targets.

#### 4.2.3. PPI Network Analysis

To acquire the Protein–Protein Interaction (PPI) Network of HS-induced myocardial damage for the subsequent identification of core targets, we imported the obtained HS-related targets into STRING 12.0, with Homo sapiens designated as the organism. The confidence level for the interaction score was set to ≥0.9. We then employed Cytoscape 3.9.1 to construct the PPI network diagram. The CytoNCA plugin in Cytoscape 3.9.1 was utilized to analyze the disease-related targets and identify the core targets using 4 algorithms: Betweenness, Closeness, Degree, and Eigenvector. Subsequently, the Molecular Complex Detection (MCODE) tool was applied for cluster analysis, with default parameters used and the MCODE score set to 4. After the analysis, the modules were visualized in a diagram according to the MCODE score.

#### 4.2.4. GO and KEGG Analyses

To investigate the molecular function (MF), biological process (BP), cellular component (CC), and pathway of common targets, we used DAVID Bioinformatics (https://david.ncifcrf.gov/tools.jsp, accessed on 15 April 2024) to perform Gene Ontology (GO) and Kyoto Encyclopedia Gene and Genome analysis (KEGG). Using bioinformatics (http://www.bioinformatics.com.cn/, accessed on 16 April 2024), the *p*-value is the lowest in the first 10 terms.

### 4.3. Molecular Docking

To determine if SA could block the pathogenic target of HS-induced myocardial damage, it was molecularly docked with IL-6 and TNF. The three-dimensional (3D) structure of the target protein, specific to Homo sapiens, was obtained from the PDB (https://www.rcsb.org/, accessed on 20 April 2024). The two-dimensional structure (2D) of SA was determined using PubChem (https://pubchem.ncbi.nlm.nih.gov/, accessed on 25 April 2024). Following the AutoDock v1.2.3 docking procedure, the binding affinity (kcal/mol) was determined. The docking box configurations were Z: 50A, Y: 50A, and X: 50A. The Number of Genetic Algorithm Runs was ten. The ligand-receptor interaction was thoroughly examined using PyMol v2.6 and LigPlot+ v2.2.8 software, and both 2D and 3D maps of the interaction were generated.

### 4.4. MD Simulation

MD simulations were used to further explore the stability and binding sites of the SA complex after docking with IL-6 and TNF. This study conducted 100 ns MD simulations of the complexes obtained from molecular docking using Gromacs v2022.03 with the CHARMM36 force field. The specific steps include converting the “pdb” format to “gro” format, adding the GAFF force field, dissolving the complex with TIP3P solvent, performing energy minimization, heating, and equilibration, and finally performing 100 ns MD simulations and saving the trajectory for analysis. Based on the obtained results, we computed the RMSD, RMSF, SASA, Rg, and H-bonds. Furthermore, we employed the “g_sham” and “xpm2txt.py” scripts in conjunction with the “MMPBSA.py v.16.0” script to determine the thermodynamic stability.

### 4.5. In Vitro Experimental Validation

#### 4.5.1. Determination of Cytotoxic Concentration of 50% (CC50)

To determine the concentration of cytotoxicity 50% (CC50) and cytotoxicity 0% (CC0) of SA on HL-1, cells were plated in 96-well plates at a density of 1 × 10^4^ cells per well. Subsequently, HL-1 cells were exposed to 8 different concentrations of SA (2000 μM, 1000 μM, 500 μM, 250 μM, 125 μM, 62.5 μM, 31.25 μM, 15.625 μM, 7.8125 μM) prepared in DMEM supplemented with 2% FBS. A control group treated with the solvent was used for comparison. After a 24 h incubation period, cell viability was assessed using the Cell Counting Kit-8 (CCK-8) assay (Vazyme; A311-01; Nanning, Guangxi, China). All procedures were performed in triplicate to ensure reproducibility. The CC50 value corresponding to each SA concentration was determined based on the assay results.

#### 4.5.2. SA Protects HL-1 Cells Against HS in a Dose-Dependent

To assess the protective effects of SA against HS in HL-1 cells, the dose-dependent treatment was conducted. In brief, HL-1 cells were seeded in 96-well plates at a density of 1 × 10^4^ cells per well. Subsequently, different concentrations of SA were added to the 96 wells at 100 μL/well, ranging from 1000 μM to 7.8125 μM. The cells were heat treated at 43 °C for 3 h, and then CCK-8 was used to evaluate the protective effect of SA on HL-1 cells.

#### 4.5.3. Target Determination by RT-qPCR

To evaluate the protective effects of SA on HL-1 cells subjected to HS, the HL-1 cells were cultured in 24-well plates at a density of 2 × 10^5^ cells per well. Subsequently, different concentrations of the SA are added to the 24 Wells. The cells were heat treated at 43 °C for 3 h to simulate HS conditions. Following this treatment, RNA extraction was performed (Vazyme; RC112; Guangxi, China), and RT-qPCR (Vazyme; Q711; Guangxi, China) was performed to determine the expression of Hsp90, Hsp70, TNF-α, IL-6, COL1A1, TLR2, MAPK3, and CDKN1A. The RNA abundance was measured with the 2^−ΔΔCt^ method. The primer sequences are shown in Table 3.

#### 4.5.4. Statistical Analysis

One-way ANOVA in GraphPad Prism (GraphPad Software; 9.5.0, Chicago, IL, USA) was used to determine significant differences (*p* < 0.05) across all experiments.

## 5. Conclusions

In summary, our study combined network pharmacology and cellular experiments to demonstrate the therapeutic potential of SA for HS-induced myocardial damage. However, a notable limitation in our study pertains to the protein-level validation of the mechanisms underlying the protective role of SA against HS-induced myocardial damage. This limitation arose from the inapplicability of certain tools, which prevented us from confirming the resistance mechanisms at the protein level. In the future, we will supplement the above deficiencies to enhance our understanding of the treatment of HS-induced myocardial damage with SA.

## Figures and Tables

**Table 1:** The number of targets collected by each database.

Keyword	Database	Number of Targets	Actual Selection	Total
Heat stress	Disgenet	3	3	2850
	CTD	7072	Top 2000	
	GeneCards	10,081	Top 2000	
	OMIM	71	71	
Cardiac injury	Disgenet	\	\	2011
	CTD	\	\	
	GeneCards	9140	Top 2000	
	OMIM	101	101	
Heart injury	Disgenet	12	12	3373
	CTD	30,393	Top 2000	
	GeneCards	10,304	Top 2000	
	OMIM	\	\	
Myocardial damage	Disgenet	\	\	2000
	CTD	\	\	
	GeneCards	5638	Top 2000	
	OMIM	57	57	

**Table 2:** Binding free energy analysis of TNF-SA and IL-6-SA complexes (kcal/mol).

Energy Contributions	TNF-SA	IL-6-SA
ΔVDWAALS	−17.23 ± 0.35	−23.72 ± 0.11
ΔEelec	−45.93 ± 0.90	−10.83 ± 2.83
ΔEGB	45.03 ± 0.49	19.62 ± 0.53
ΔEsurf	−3.65 ± 0.00	−3.73 ± 0.01
ΔGgas	−63.17 ± 0.97	−34.56 ± 2.83
ΔGsolvation	41.38 ± 0.49	15.89 ± 0.53
ΔGBind	−21.79 ± 1.08	−18.67 ± 2.88

**Table 3:** The primer sequences of RT-qPCR.

	Primer Sequence
Hsp90	F: 5′-GTGTGCAACAGCTGAAGGAA-3′
	R: 5′-ACAGCAGCACTGGTGTCATC-3′;
Hsp70	F: 5′-CGACCTGAACAAGAGCATCA-3′
	R: 5′-ATGACCTCCTGGCACTTGTC-3′
TNF-α	F: 5′-CACGTCGTAGCAAACCACCAA-3′
	R: 5′-GTTGGTTGTCTTTGAGATCCAT-3′
IL-6	F: 5′-CCACTTCACAAGTCGGAGGCTTA-3′
	R: 5′-CCAGTTTGGTAGCATCCATCATTTC-3′
COL1A1	F: 5′-GCTCCTCTTAGGGGCCACT-3′
	R: 5′-CCACGTCTCACCATTGGGG-3′
TLR2	F: 5′-GCAAACGCTGTTCTGCTCAG-3′
	R: 5′-AGGCGTCTCCCTCTATTGTATT-3′
MAPK3	F: 5′-TCCGCCATGAGAATGTTATAGGC-3′
	R: 5′-GGTGGTGTTGATAAGCAGATTGG-3′
CDKN1A	F: 5′-TACGGCAACACTGGGTAACC-3′
	R: 5′-GACCATCTGGGGTGGT-3′

## Data Availability

The original contributions presented in the study are included in the article, further inquiries can be directed to the corresponding authors.

## Supplementary Materials

The following supporting information can be downloaded at: https://www.mdpi.com/article/10.3390/ph17111485/s1, Table S1. Details for the enrichment analysis of cellular component, molecular function and biological process of 60 common targets and the KEGG pathway analysis (Figure 3C–F).
